# Should lower respiratory tract secretions from intensive care patients be systematically screened for influenza virus during the influenza season?

**DOI:** 10.1186/cc11387

**Published:** 2012-06-14

**Authors:** Maddalena Giannella, Belen Rodríguez-Sánchez, Paula López Roa, Pilar Catalán, Patricia Muñoz, Darío García de Viedma, Emilio Bouza

**Affiliations:** 1 Department of Clinical Microbiology and Infectious Diseases, Hospital General Universitario Gregorio Marañón, Doctor Esquerdo 46, Madrid, 28007, Spain; 2 CIBER de Enfermedades Respiratorias (CIBERES), Recinto Hospital Joan March Street 2: Crta. Soller, Km 12, Bunyola, Mallorca 07110, Spain; 3 Department of Medicine, Universidad Complutense de Madrid, Av de Puerta de Hierro, Madrid, 28040, Spain

## Abstract

**Introduction:**

Influenza is easily overlooked in intensive care units (ICUs), particularly in patients with alternative causes of respiratory failure or in those who acquire influenza during their ICU stay.

**Methods:**

We performed a prospective study of patients admitted to three adult ICUs of our hospital from December 2010 to February 2011. All tracheal aspirate (TA) samples sent to the microbiology department were systematically screened for influenza. We defined influenza as unsuspected if testing was not requested and the patient was not receiving empirical antiviral therapy after sample collection.

**Results:**

We received TA samples from 105 patients. Influenza was detected in 31 patients and was classified as unsuspected in 15 (48.4%) patients, and as hospital acquired in 13 (42%) patients. Suspected and unsuspected cases were compared, and significant differences were found for age (53 versus 69 median years), severe respiratory failure (68.8% versus 20%), surgery (6.3% versus 60%), median days of ICU stay before diagnosis (1 versus 4), nosocomial infection (18.8% versus 66.7%), cough (93.8% versus 53.3%), localized infiltrate on chest radiograph (6.3% versus 40%), median days to antiviral treatment (2 versus 9), pneumonia (93.8% versus 53.3%), and acute respiratory distress syndrome (75% versus 26.7%). Multivariate analysis showed admission to the surgical ICU (odds ratio (OR), 37.1; 95% confidence interval (CI), 2.1 to 666.6; *P *= 0.01) and localized infiltrate on chest radiograph (OR, 27.8; 95% CI, 1.3 to 584.1; *P *= 0.03) to be independent risk factors for unsuspected influenza. Overall mortality at 30 days was 29%. ICU admission for severe respiratory failure was an independent risk factor for poor outcome.

**Conclusion:**

During the influenza season, almost one third of critical patients with suspected lower respiratory tract infection had influenza, and in 48.4%, the influenza was unsuspected. Lower respiratory samples from adult ICUs should be systematically screened for influenza during seasonal epidemics.

## Introduction

Influenza is a common cause of admission to the intensive care unit (ICU) during the influenza season and influenza pandemics [1-4]. However, it may be overlooked, particularly in patients with clinical manifestations that can be explained by alternative infectious or noninfectious causes [5]. Furthermore, influenza may not be suspected when respiratory function deteriorates or fails in patients already admitted to the ICU.

At present, information on influenza acquired during ICU stay is scarce and incomplete [5]. Timely knowledge of the presence of influenza virus in patients admitted to the ICU has obvious epidemiologic, diagnostic, and therapeutic advantages [4].

We assessed the burden of influenza in adult ICUs and the number of overlooked cases when the routine diagnostic workup was applied during the influenza season. We screened all tracheal aspirates sent to the microbiology department for the diagnosis of lower respiratory tract infection, even when not requested by the attending physician.

## Materials and methods

### Setting

Our hospital is a 1,550-bed tertiary referral teaching institution caring for a population of approximately 750,000 inhabitants. It has three different adult ICUs (medical, surgical, and cardiac surgery) with a total of 42 beds.

### Design

From December 15, 2010, through February 28, 2011, all tracheal aspirate (TA) samples obtained from adult patients (≥18 years) admitted to our ICUs and sent to the microbiology department were systematically screened for influenza virus.

ICU admission criteria and management for all patients, including the need for intubation and for obtaining TA samples, were not standardized, and decisions were made at the discretion of the attending physician.

Patients with laboratory-confirmed influenza, by real-time reverse transcriptase polymerase chain reaction (RT-PCR) on TA and nasopharyngeal samples, were prospectively followed up by an infectious diseases specialist and treated with oseltamivir, 150 mg/day, for 5 to 10 days. Clinical and microbiology data were recorded in a preestablished protocol and entered into a database.

The study was approved by the ethics committee of the "Fundación para la Investigación Biomédica del Hospital Gregorio Marañón." The requirement for informed consent was waived because we applied an excellent diagnostic technique to improve the quality of patient care without any negative impact.

Our objectives were to determine the incidence of influenza among adult ICU patients with a TA sample obtained during the influenza season, and to demonstrate the frequency of unsuspected cases and the rate of hospital-acquired episodes.

### Data collected

The variables recorded were age, sex, classification of the severity of underlying conditions according to the Charlson comorbidity index [6], type of ICU, date and cause of ICU admission, APACHE II score [7] on admission to the ICU, date of onset of influenza symptoms, clinical manifestations and radiologic findings at diagnosis, date of TA sample collection, other samples tested for influenza and result, date of initiation of antiviral treatment, complications (septic shock, acute respiratory distress syndrome (ARDS)), outcome including mortality within 30 days after influenza diagnosis, and length of ICU and hospital stay.

### Definitions

We defined the diagnosis of influenza as unsuspected when influenza testing was not explicitly requested or had not been previously requested in other samples, such as nasopharyngeal swabs, and the patient was not receiving empirical antiviral treatment immediately after sample collection.

Influenza was classified as community acquired if the flu syndrome (fever, chills, malaise, sore throat, rhinorrhea, cough, dyspnea, myalgia, nausea, and diarrhea) began before or during the first 72 hours of hospital admission. The infection was classified as hospital-acquired, if symptoms started after the first 72 hours [8].

As for causes of ICU admissions, severe respiratory failure was defined as severe hypoxemia (PaO_2 _< 60 mm Hg) refractory to high-flow oxygen therapy (FiO_2_, 50%) with a Venturi mask.

As for underlying conditions, chronic obstructive pulmonary disease was defined according to the criteria of the 2007 Global Initiative for Chronic Obstructive Lung Disease [9]. Immunosuppressed patients were those with hematologic malignancy (with or without bone marrow transplantation), HIV infection, inflammatory diseases under biologic or immunosuppressive treatment and solid organ transplant. As for influenza vaccination, we considered patients who had been vaccinated against influenza within 6 months before admission.

Pneumonia was defined according to the current IDSA/ATS guidelines [10]. ARDS and septic shock were defined by using standard criteria [11,12].

### Microbiologic procedures

Samples for microbiologic diagnosis were taken by endotracheal aspiration with a 14F sterile probe to a depth of 2 cm from the distal end of the endotracheal tube. The secretions obtained were collected in a sterile container (Lukens Specimen Container; Sherwood Medical, Tullamore, Ireland) and transported in sterile packages to the microbiology laboratory for Gram staining and bacterial and viral procedures.

Standard bacterial procedures included quantitative culture performed on blood agar, chocolate agar, McConkey agar, and, when required, *Legionella *agar (BCYE) [13]. Positive samples were defined as those with bacterial counts ≥10^5 ^cfu/ml of each significant microorganism. The microorganisms were identified and antimicrobial susceptibility testing performed by using an automatic system (MicroScan; Dade Behring, Sacramento, CA, USA). Breakpoints were determined after the Clinical and Laboratory Standards Institute (CLSI) guidelines [14]. Unless proven otherwise, we considered as nonpathogenic the isolation (at any concentration) of the following microorganisms: *viridans*-group streptococci, *Enterococcus *spp., coagulase-negative *Staphylococcus*, *Neisseria *spp., *Corynebacterium *spp., and *Candida *spp.

Samples were collected in viral-transport medium (Copan 305C; Copan Innovation, Brescia, Italy). A 200-μl aliquot was stored at 4°C for no longer than 48 hours until analysis. The rest of the sample was stored at -80°C for further amplification and sequencing.

RNA was extracted in a Nuclisens EasyMAG system (BioMérieux, Boxtel, The Netherlands) by following the manufacturer's instructions. Pandemic influenza A pH1N1 was detected by real-time reverse transcriptase polymerase chain reaction (RT-PCR) by following the WHO/CDC protocol in a Stratagene MX3000 thermocycler (Stratagene, La Jolla, CA, USA). Those samples rendering indeterminate results (low-fluorescence signal or high Ct values) were tested again with the RealTime ready Inf A/H1N1 Detection Set (Roche Diagnostics, Mannheim, Germany). Influenza B was detected by using the RealTime ready Influenza B Detection Set (Roche Diagnostics). H3N2 and seasonal H1N1 strains were detected as described elsewhere [15].

Relative DNA was quantified by combining the RT-PCR methods described with the detection of a housekeeping gene with real-time RT-PCR, as described by the CDC. This method allowed normalization of the initial amount of RNA present in each sample [16].

### Statistical analysis

Categoric variables appear with their frequency distribution. Nonnormally distributed continuous variables are expressed as the median and interquartile range (IQR). The association between categoric variables was evaluated by using the χ^2 ^test or Fisher Exact test; the association between continuous variables was evaluated by using the Mann-Whitney *U *test.

A logistic binary model was used to analyze the independent risk factors for unsuspected influenza and 30-day mortality. Variables with *P *≤ 0.1 in the univariate analysis were entered into the multivariate model. The level of significance was set at *P *< 0.05 for all the tests. The statistical analysis was performed by using SPSS 13.0.

## Results

**Figure 1:**
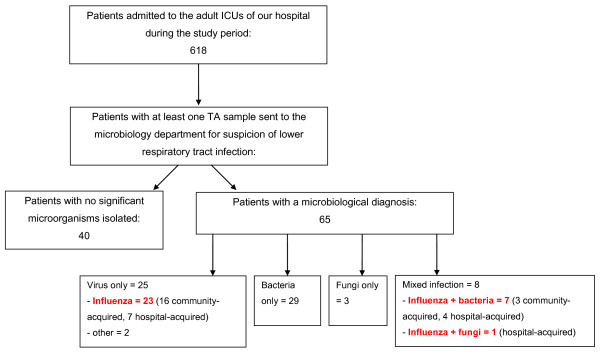
**Flow diagram of study patients**. ICU, intensive care unit; TA, tracheal aspirate.

During the study period, 618 patients were admitted to our adult ICUs. Overall, one or more TA samples were obtained from 105 patients, and a microbiologic diagnosis was made in 65 of them (see Figure 1). Bacterial infection was diagnosed in 29 patients, and the frequencies of the pathogens isolated were as follows: *Staphylococcus aureus*, 37.9%; Enterobacteriaceae, 24.1%; *Pseudomonas aeruginosa*, 17.2%; *Streptococcus pneumoniae*, 13.7%; and *Acinetobacter baumannii*, 6.8%. A diagnosis of viral infection only was made in 25 patients: 23 with influenza virus, one with adenovirus, and one with herpes simplex virus. *Aspergillus fumigatus *was the only microorganism isolated in three patients. The remaining eight patients initially had coinfection with influenza virus and the following microorganisms: *S. aureus*, three; *S. pyogenes*, one; *S. pneumoniae*, one; *A. baumannii*, one; *P. aeruginosa*, one; and *Aspergillus fumigatus*, one.

### Incidence and clinical characteristics of patients with influenza

During the study period, the overall incidence of influenza in the adult ICUs of our hospital was 5.3 cases per 100 ICU admissions. The incidence of influenza among the patients with at least one TA sample sent to the microbiology department was 29.5 cases per 100 ICU patients.

The reasons for admission to the ICU and the characteristics and outcome of the 31 patients with influenza are shown in Table 1. Influenza was unsuspected in 15 (48.4%) patients and hospital-acquired in 13 (42%) patients. At the time of influenza diagnosis, all patients but one were intubated.

**Table 1:** Characteristics of ICU patients diagnosed with influenza

	*n *= 31 (%)
Demographic data	
Age (years) (median, IQR)	64, 48-70
Male sex	20 (64.5)

Type of ICU	
Medical	21 (67.7)
Surgical	10 (32.3)

Reason for admission to the ICU	
Severe respiratory failure	14 (45.1)
Surgery	10 (32.2)
Cardiac arrest	4 (12.9)
Other^a^	3 (9.6)
APACHE II score	15, 12-17

Characteristics of influenza	
Days of symptoms before diagnosis (median, IQR)	3, 0-7
Fever	22 (71)
Cough	23 (74.2)
Dyspnea	24 (77.4)
Diarrhea	3 (9.7)
Pneumonia	23 (74.2)
Co-infection	8 (25.8)
Unsuspected infection	15 (48.4)
Hospital-acquired infection	13 (42)

Influenza virus	
H1N1v	27 (87)
B	3 (9.7)
H3N2	1 (3.2)

Complications	
Septic shock	13 (41.9)
ARDS	16 (51.6)
30-day mortality	9 (29)

^a^Acute hepatic failure (one patient), necrotizing pancreatitis (one patient), and decompensated cirrhosis (one patient). ARDS, acute respiratory distress syndrome; ICU, intensive care unit; IQR, interquartile range.

Among patients with co-infection, the reasons for admission to the ICU were as follows: surgery, five; respiratory failure, one; cardiac arrest, one; and decompensated cirrhosis, one. Influenza was classified as hospital acquired in five (62.5%) of them, and pneumonia was diagnosed in seven (87.5%) patients.

### Microbiologic characteristics

Overall, viral infection was diagnosed in 33 patients, and in 31 (93.9%) of them, influenza was detected. Influenza was due to the 2009 pandemic influenza A H1N1 strain in 27 (87%) patients, influenza B in three (9.7%) patients, and influenza A H3N2 in one (3.2%) patient.

In 17 of the 31 patients, influenza testing was performed simultaneously in the TA and nasopharyngeal samples. The upper respiratory tract sample failed to detect influenza in 17.6% of cases.

Overall, the median relative viral load at diagnosis was 1.55 (IQR, 0.68 to 3.16). This tended to be higher in patients with suspected influenza (Table 2).

**Table 2:** Comparison of patients with suspected and unsuspected influenza

	Suspected	Unsuspected	*P*
	*n *= 16 (%)	*n *= 15 (%)	
Demographic data			
Age (years) (median, IQR)	53, 42-66	69, 60-79	0.008
Male sex	11 (68.8)	9 (60)	0.71

Underlying conditions			
COPD	7 (43.8)	7 (46.7)	1
Immunosuppression	3 (18.8)	2 (13.3)	1
Charlson score	4.5, 2.2-7	6, 3-9	0.26
Influenza vaccination	4 (25)	3 (20)	1

Type of ICU			
Medical	15 (93.8)	6 (40)	0.002
Surgical	1 (6.3)	9 (60)	

Reason for admission to the ICU			
Severe respiratory failure	11 (68.8)	3 (20)	0.002
Surgery	1 (6.3)	9 (60)	
Cardiac arrest	3 (18.8)	1 (6.7)	
Other	1 (6.3)	2 (13.3)	
APACHE II score	15.5, 12.5-17.5	14, 11-17	0.40

Characteristics of influenza			
Days of symptoms before diagnosis^a^	3, 1-6.7	5, 0-9	0.92
Days of ICU stay before diagnosis^a^	1, 0-1	4, 1-17	0.01
Days of hospital stay before diagnosis^a^	2.50, 1-5.75	15, 5-22	0.008
Hospital acquired	3 (18.8)	10 (66.7)	0.01
Fever	11 (68.8)	11 (73.3)	1
Cough	15 (93.8)	8 (53.3)	0.01
Dyspnea	13 (81.3)	11 (73.3)	0.68
Pneumonia	15 (93.8)	8 (53.3)	0.01
Localized infiltrate on chest radiograph	1 (6.3)	6 (40)	0.04
Viral load at diagnosis (median, IQR)	2.37, 1.10-5.42	1.4, 0.23-1.97	0.09
Days to antiviral treatment^a^	2, 2-6	9, 4.5-18	0.02
Co-infection	3 (18.8)	5 (33.3)	0.43

Outcome			
Septic shock	8 (50)	5 (33.3)	0.47
ARDS	12 (75)	4 (26.7)	0.01
30-day mortality	6 (37.5)	3 (20)	0.43
Days of ICU stay (median, IQR)	11, 4-33	27, 8-50	0.21
Days of hospital stay (median, IQR)	39, 4-46	66, 30-90	0.06

^a^Variables are expressed as median and interquartile range. ARDS, acute respiratory distress syndrome; COPD, chronic obstructive pulmonary disease; ICU, intensive care unit; IQR, interquartile range.

### Comparison of suspected and unsuspected cases

Patients with suspected influenza were compared with those with unsuspected influenza (Table 2). The univariate analysis revealed significant differences for age (53 versus 69 years; *P *= 0.008), medical ICU (93.8% versus 40%; *P *= 0.002), admission to the ICU for severe respiratory failure (68.8% versus 20%; *P *= 0.002), length of ICU stay before the influenza diagnosis (1 (IQR, 0 to 1) versus 4 (IQR, 1 to 17) days; *P *= 0.01), classification as having hospital-acquired influenza (18.8% versus 66.7%; *P *= 0.01), cough (93.8% versus 53.3%; *P *= 0.01), localized pulmonary infiltrate on radiograph (6.3% versus 40%; *P *= 0.04), median days to initiation of antiviral therapy after onset of symptoms (2 (IQR, 2 to 6) versus 9 (IQR, 4.5 to 18) days; *P *= 0.02), pneumonia (93.8% versus 53.3%; *P *= 0.01), and development of ARDS (75% versus 26.7%; *P *= 0.01). Mortality at 30 days after the influenza diagnosis was 37.5% and 20% (*P *= 0.43) in patients with suspected and unsuspected influenza, respectively.

Multivariate analysis showed the independent risk factors associated with unsuspected influenza to be admission to the surgical ICU (OR, 37.13; 95%CI, 2.06 to 666.60; *P *= 0.01) and localized pulmonary infiltrate on radiograph (OR, 27.78; 95%CI, 1.32 to 584.06; *P *= 0.03). Longer ICU stay before the diagnosis of influenza was also associated with unsuspected influenza but was not significant (Table 3).

**Table 3:** Multivariate analysis of risk factors for unsuspected influenza

	Adjusted odds ratio (95% CI)	*P*
Surgical ICU	37.13 (2.06-666.60)	0.01
Localized infiltrate on chest radiograph	27.78 (1.32-584.06)	0.03
Days of ICU stay before influenza diagnosis	1.31 (0.97-1.78)	0.07

CI, confidence interval; ICU, intensive care unit.

### Outcome

Overall mortality at 30 days after influenza diagnosis was 29%. The univariate analysis of the risk factors for mortality is shown in Table 4. Nosocomial acquisition of influenza was associated with better outcome (54.5% versus 11.1%; *P *= 0.04). The only independent risk factor for 30-day mortality in the multivariate analysis was severe respiratory failure as the reason for admission to the ICU (OR, 7.5; 95%CI, 1.23 to 45.8; *P *= 0.03).

**Table 4:** Univariate analysis of risk factors for 30-day mortality

	Survivors	Nonsurvivors	*P*
	*n *= 22 (%)	*n *= 9 (%)	
Demographic data			
Age (years) (median, IQR)	66.5, 57.7-75.5	54, 43-62	0.09
Male sex	15 (68.2)	5 (55.6)	0.68

Underlying conditions			
Immunosuppression	4 (18.2)	1 (11.1)	1
COPD	9 (40.9)	5 (55.6)	0.69
Charlson score	6, 2-8	5, 3.5-6.5	0.65
Influenza vaccination	5 (22.7)	2 (22.2)	1

Type of ICU			
Medical	13 (59.1)	8 (88.9)	0.20
Surgical	9 (40.9)	1 (11.1)	

Reason for admission to the ICU			
Severe respiratory failure	7 (31.8)	7 (77.8)	0.08
Surgery	9 (40.9)	1 (11.1)	
Cardiac arrest	4 (18.2)	0	
Other	2 (9.1)	1 (11.1)	
APACHE II score	14.5, 11-16.2	16, 13-19.5	0.22

Characteristics of influenza			
Days of symptoms before diagnosis^a^	3.5, 0-9	3, 1.5-5	0.87
Days of ICU stay before diagnosis^a^			
Unsuspected diagnosis	1, 0-14.5	1, 0-3	0.26
Hospital acquired	12 (54.5)	3 (33.3)	0.43
Fever	12 (54.5)	1 (11.1)	0.04
Cough	15 (68.2)	7 (77.8)	0.68
Dyspnea	14 (63.6)	9 (100)	0.07
Localized infiltrate on chest radiograph	15 (68.2)	9 (100)	0.07
Viral load at diagnosis	4 (18.2)	3 (33.3)	0.64
Pneumonia	1.5, 0.3-2.4	1.8, 0.8-5.5	0.40
Co-infection	15 (68.2)	8 (88.9)	0.38
	6 (27.3)	2 (22.2)	1

^a^Variables are expressed as median and interquartile range. COPD, chronic obstructive pulmonary disease; ICU, intensive care unit; IQR, interquartile range.

## Discussion

During the influenza season, almost one third of patients hospitalized in our adult ICUs and with suggestion of lower respiratory tract infection had influenza. Influenza was unsuspected in 48.4% and hospital acquired in 42%. Patients with unsuspected influenza were more frequently admitted to the ICU for surgery, had a localized infiltrate on chest radiograph, and stayed longer in the ICU before being diagnosed with influenza. Antiviral treatment was initiated later in patients with unsuspected influenza, although mortality was similar in both groups. Overall mortality at 30 days after the influenza diagnosis was 29%; however, it was lower in patients with nosocomial influenza. Severe respiratory failure as the cause of admission to the ICU was the only independent factor associated with poor outcome.

Acute febrile respiratory illness is a common cause of respiratory failure and admission to the ICU [2-4]. In most cases, the etiology is bacterial, although viruses have been implicated in almost 9% of cases [17]. During the 2009 pandemic, the rate of ICU admission for respiratory failure among hospitalized patients with a confirmed diagnosis of influenza A (H1N1v) ranged from 15% to 34% [18-22]. However, no studies have investigated the rates of bacterial and viral etiologies among patients admitted to the ICU with suggestion of lower respiratory tract infection during the 2009 pandemic. Here, we demonstrated that, after the pandemic influenza season, the etiology was viral in 31.4% of patients admitted to the ICU with suggestion of lower respiratory tract infection. Influenza was detected in most of these cases (93.9%).

The etiology of acute febrile respiratory illness causing respiratory failure is often unknown at admission to the ICU [17]. About half of the cases are diagnosed as bacterial pneumonia shortly after admission, with a small number of cases found to be viral pneumonia when the initial bacterial studies are negative [10]. Detection of influenza virus often depends on specific epidemiologic risk factors and clinical suspicion. The combination of fever, malaise, and cough was shown to have a 79% positive predictive value during the pandemic and seasonal epidemics [23,24]; however, these criteria may be not accurate in ICU patients, because other etiologies, or conditions like as postsurgery sedation, may confound the diagnosis [25]. In our study, influenza was unsuspected in 48.4% of cases. Suspicion of influenza was lower in older patients, in those admitted to the ICU for surgical conditions, in those who stayed for a longer time in hospital and ICU, and in those who did not have a cough and diffuse pulmonary infiltrates. The direct consequence of overlooked influenza was a significant delay in the initiation of antiviral treatment.

Definitive diagnosis of influenza is by detection of the virus in culture or RT-PCR with a nasopharyngeal aspirate/swab or lower respiratory tract sample [23,24]. Because viral shedding peaks at 48 hours after the onset of illness and declines thereafter, testing of lower respiratory tract samples in patients with compromised lung parenchyma may be more beneficial [23,26,27]. Accordingly, we found that the upper respiratory tract sample did not reveal influenza in 17.6% of cases. Diagnostic viral load tended to be higher in patients with suspected influenza, possibly as a result of the earlier diagnosis of influenza after onset of symptoms in this group compared with patients with unsuspected influenza.

Hospital-acquired influenza is a well-recognized problem [28,29]. Nosocomial outbreaks of pandemic and seasonal influenza have been documented in various settings, including ICUs, pediatric wards, transplant units, medical wards, and surgical wards [28-32]. However, few sporadic cases of hospital-acquired influenza have been reported during surveillance activities [33]. In a study including 1,520 patients hospitalized with the pandemic 2009 influenza A in 75 hospitals in the United Kingdom, the authors identified 30 (2%) cases of sporadic nosocomial influenza [33]. These comprised 15 adults and 15 children. Most had serious underlying illnesses and were admitted to nonmedical areas, as in our study. Unexpectedly, we found that the 30-day mortality rate was lower in patients with hospital-acquired influenza. This figure can be associated with viral factors, such as lower virulence of the influenza strains circulating in the hospital, or with host factors, such as older age and surgical conditions.

Overall, 30-day mortality was high (29%), and admission to the ICU for severe respiratory failure was an independent risk factor for death. These data are consistent with those of Martin-Loeches *et al. *[34], who showed that patients from the postpandemic influenza pH1N1 period had an unexpectedly high mortality rate. Early administration of antiviral therapy has been associated with better outcome in critically ill patients [35]. In our study, although the timing to initiation of antiviral treatment was longer among patients with unsuspected influenza, a trend to lower mortality was seen in this group compared with patients with suspected influenza. A possible explanation of this finding could be that: suspected and unsuspected groups were epidemiologically very different, and the median relative viral load was lower in the unsuspected group; thus, epidemiologic and viral factors could influence the outcome in the two groups independently of the timing of antiviral treatment. Conversely, the benefit of testing will not be necessarily to the patient in terms of improved outcome due to early therapy, but more likely to preventing the nosocomial transmission of influenza.

Our study is limited in that the small number and heterogeneity of patients diminishes the power of our data analysis. We performed the study during the postpandemic period (2010 to 2011), when the prevalence of the pandemic influenza A H1N1 strain was still high. Findings could vary between one influenza season and another, depending on the characteristics of the prevalent influenza virus stain. We did not perform a cost-effectiveness analysis, although the finding of a longer ICU and hospital stay in patients with unsuspected influenza suggests a potential favorable impact on care management. We could not perform an analysis of the possible routes of transmission of the nosocomial cases. However, we can exclude with sufficient certainty the occurrence of an outbreak for the following reasons: (a) the cases of hospital-acquired influenza were distributed uniformly between the three ICUs (postsurgery ICU, six; medical ICU, five; and postcardiosurgery ICU, two); (b) no case of influenza was recognized among the health-care staff during the study period; (c) the preventive measures included vaccination of staff, respiratory isolation, and droplet-contact precautions, as recommended by the Centers for Disease Control and Prevention [36].

## Conclusions

We showed that influenza is a common cause of acute respiratory illness among patients admitted to the ICU during seasonal epidemics, and that it is often overlooked, and it could lead to a delay in the initiation of antiviral treatment and possible nosocomial transmission of influenza.

Microbiology departments should systematically investigate the presence of influenza in respiratory samples obtained from ICU patients during the seasonal epidemic.

## Key messages

• The incidence of influenza in the adult ICU during the influenza season is high.

• The diagnosis of influenza is often overlooked in ICU patients. Among patients with unsuspected influenza, the timing to initiation of antiviral treatment was longer, and the rate of hospital-acquired influenza was higher compared with that of patients with suspected influenza.

• Microbiology departments should systematically investigate the presence of influenza in respiratory samples obtained from ICU patients during the seasonal epidemic.

## Abbreviations

APACHE: acute physiology and chronic health evaluation; ARDS: acute respiratory distress syndrome; ATS: American Thoracic Society; CDC: Centers for Disease Control; COPD: chronic obstructive pulmonary disease; ICU: intensive care unit; IDSA: Infectious Diseases Society of America; IQR: interquartile range; RT-PCR: reverse transcriptase-polymerase chain reaction; TA: tracheal aspirate; WHO: World Health Organization.

## Competing interests

The authors declare that they have no competing interests.

## Authors' contributions

All the authors made a substantial contribution. EB, DGdV, PC, and PM assisted in the conception and design of the study, revised the manuscript critically, and gave the final approval of the version to be published. MG, BR, and PLR were responsible for data acquisition, analysis, and interpretation. MG drafted the manuscript. Members of GANG study group revised and approved the study design and assisted in the data acquisition. All authors read and approved the final manuscript.
